# Bias in Amputation Research; Impact of Subjects Missed from a Prospective Study

**DOI:** 10.1371/journal.pone.0043629

**Published:** 2012-08-20

**Authors:** Lauren V. Fortington, Jan H. B. Geertzen, Joline C. Bosmans, Pieter U. Dijkstra

**Affiliations:** 1 Department of Rehabilitation Medicine, University Medical Center Groningen, Groningen, The Netherlands; 2 Department of Oral and Maxillofacial Surgery, University Medical Center Groningen, Groningen, The Netherlands; Sapienza University of Rome, Italy

## Abstract

For research findings to be generalized, a sample must be representative of the actual population of interest. Lower limb amputation is most frequently performed in older patients with vascular disease, a population that is often under-represented in research. The aim of this study was to explore the impact of selection bias by comparing characteristics from a sample included in a prospective study of phantom pain with the actual population who underwent amputation. Only 27% of all potential patients were referred during the first year of the prospective study. The referred patients were 8 years younger (p<0.001) and less likely to have had amputation because of a vascular condition, diabetes or infection (p = 0.003) than those not referred. There was also a significant difference in one year survival between the groups; 67% of referred patients survived compared with just 40% of non-referred patients (p = 0.004). The biased population in the phantom pain study may have resulted in an underestimation of phantom pain in the original study and subsequent protective factors should be considered within the context of the younger population reported. Selection bias is common in amputation research, and research methods to minimize its impact must be given greater attention.

## Introduction

After a lower limb amputation (LLA), people face a number of challenges including loss of mobility, altered body image and phantom pain. Research to better understand these consequences allows planning for rehabilitation and long term care and builds an evidence base from which we can more accurately inform patients on their expected outcomes. However, difficulties with population sampling are frequent in amputation research and this impacts our ability to draw accurate conclusions.

LLA is most frequently performed in older patients with vascular disease, a population that is, for the most part, under-represented in research [1]. Multiple co-morbidities and cognitive decline can prevent this sub-population from meeting required inclusion criteria. The issue of bias in studies of elderly people is well recognized [1]–[4]. The same is true of amputation research, with authors’ invariably describing selection bias within their sample as a limitation of their study [5]–[11]. However, the impact of this bias is rarely described [12]. It is important to understand this research limitation in applying results to clinical practice and to better design future studies.

In a prospective study of phantom limb pain, it was noted that the population characteristics of the included sample were considerably different from what would be expected in the LLA population [5]. The aim of the current study is to explore the impact of this bias on the primary outcomes (factors associated with phantom pain) by comparing the study sample with the actual population who underwent amputation.

## Methods

### Ethics Statement

The study protocol was approved by the medical ethics committees of the University Medical Center Groningen. Patients participating in the phantom pain study provided their written informed consent.

### Setting

Both studies were conducted in the 3 Northern provinces of the Netherlands: Groningen, Friesland and Drenthe. Fourteen hospitals in the region performed lower limb amputations, generally under the care of a vascular surgeon. This study looks at patients who had a first ever unilateral transtibial amputation, knee disarticulation or transfemoral amputation between 1 January 2004 and 31 December 2004.

### Phantom Pain (PP) Study

A prospective study ran from 1 November 2003 to 30 April 2008. At a face-to-face meeting, and confirmed afterwards in writing, surgeons were informed about the study including the aims, inclusion and exclusion criteria, and recruitment procedures.

The surgeons were requested to include all patients: (a) aged ≥18 years; (b) undergoing primary major amputation (at or proximal to metatarsophalangeal level); and (c) able to read and write in Dutch. The primary investigator discussed the study aims with the patient and they were asked to participate and give their written consent.

Exclusion criteria were: (a) had a previous ipsilateral amputation; (b) were too unwell or showed signs of clinical dementia which prevented completion of the questionnaires, or (c) were recruited more than 5 days after the amputation.

If the surgeons themselves decided to exclude a patient, they agreed to send the characteristics of the patient (age, sex) and amputation details (level, cause) to the primary investigator to ensure a complete census of patients was recorded. The primary investigator maintained regular contact with the study coordinator at each hospital.

### Population Study

In 2010, surgeons from each hospital were contacted about a new study on the incidence of LLA. Surgeons from all hospitals agreed to participate. They were requested to compile a list of patients who underwent major amputation in 2004. The medical records of these patients were reviewed between August 2010 and July 2011 for patient data (age, sex), amputation details (level, cause), marital status, comorbidities and medical history including previous minor amputations or peripheral vascular procedures (angioplasty, embolectomy or peripheral bypass) and survival or date of death. To ensure a complete survival dataset, general practitioners were contacted for patients whose status was not up to date in hospital records.

### Statistical Analysis

Characteristics of referred patients (irrespective of whether they were included or excluded from analyses in the original study) were compared with the non-referred patients using chi-square tests for categorical variables, Mann-Whitney U test for age distribution and log rank tests for survival. Significance was set at 0.05 and analyses were performed using PASW Statistics version 18.0.

## Results

Surgeons representing 12 of 14 hospitals attended the information meeting of the PP study. Two hospitals were unable to participate because of restrictions from their local administration and medical ethics procedures. Surgeons from ten hospitals agreed to participate in recruitment of patients.

From the current study, one hospital was unable to identify the relevant files because of changes in their database. This hospital was excluded and subsequently, one patient from this hospital who had been referred to the PP study was excluded.

Thirty nine (27%) of a possible 146 patients were referred during the first full year of the study (table 1). The referred population had a median age 8 years younger (p<0.001) and were less likely to have had amputation because of a vascular condition, diabetes or infection (p = 0.003) than those who were not referred. More non-referred patients had bilateral amputation while more referred patients had a knee disarticulation (p = 0.049). No differences in the number or type of major co-morbidities were seen, although referred patients were more likely to have had undergone a previous vascular intervention (p = 0.042) such as a peripheral bypass procedure or angioplasty. Referred patients were more frequently discharged home or to a rehabilitation centre with non-referred patients more often discharged to a care centre (p = 0.020).

**Table 1:** Characteristics of referred and non-referred patients and actual population.

n (%), unless stated otherwise	Referred (PP study)		Not referred		p	Actual population	
Total included	39	(27)	107	(73)		146	
Age, median (IQR)*	67.6	(50.8; 72.9)	75.5	(68.1; 83.3)	<0.001†	73.0	(65.2; 80.9)
Age, mean (sd)	63.0	(13.9)	74.4	(12.0)	<0.001‡	71.4	(13.5)
Men	24	(62)	63	(59)	0.463	87	(60)
*Cause of amputation*							
Vascular	31	(80)	101	(96)	0.003	132	(92)
Other	8	(21)	4	(4)		12	(8)
*Level of amputation*							
bilateral	2	(5)	11	(10)	0.049§	13	(9)
transfemoral	12	(32)	32	(30)		44	(30)
knee disarticulation	7	(18)	5	(5)		12	(8)
transtibial	17	(45)	59	(55)		76	(52)
*Admitted from*							
home	24	(75)	58	(62)	0.125	82	(65)
care	8	(25)	36	(38)		44	(35)
*Marital status*							
married/partnership	21	(64)	42	(49)	0.118	63	(53)
single/widow/divorced	12	(36)	43	(51)		55	(47)
*Number of comorbidities*							
0	7	(23)	9	(10)	0.171	16	(13)
1–2	15	(48)	56	(63)		73	(59)
≥3	9	(29)	24	(27)		33	(28)
*Type of comorbidities*							
peripheral vascular disease	21	(57)	54	(51)	0.320	75	(52)
hypertension	16	(42)	39	(36)	0.334	55	(38)
diabetes	14	(37)	45	(42)	0.358	59	(41)
congestive heart failure	6	(16)	26	(24)	0.217	32	(22)
myocardial infarct	5	(13)	14	(13)	0.593	19	(13)
cerebrovascular disease	3	(8)	17	(16)	0.185	20	(14)
chronic lung disease	8	(22)	18	(17)	0.335	26	(18)
kidney disease	9	(24)	21	(20)	0.376	30	(21)
Peripheral vascular procedure	23	(59)	44	(41)	0.042	67	(46)
*Discharged to*							
home	10	(26)	16	(16)	0.020	26	(18)
rehabilitation centre	9	(23)	8	(8)		17	(12)
care	15	(39)	53	(52)		68	(48)
died before discharge	5	(13)	25	(25)		30	(21)
12 month survival	26	(67)	43	(40)	0.004	69	(47)
*Hospital* **>10 amputations	29	(27)	80	(73)	0.960	109	(75)
≤10 amputations	10	(27)	27	(73)		37	(25)

p is difference between referred and non-referred groups;

* Median age presented because data were not normally distributed, and mean age also presented to enable comparison to original PP Study;

† Mann Whitney U Test;

‡ Independent sample t-test; all others are Chi-square test;

§ Exact method used as cell count assumptions not met;

** Comparison of hospitals where there were >10 (n = 6) amputations with ≤10 amputations (n = 7); Actual population is presented to enable comparison of characteristics, no statistical analysis was performed; not all percentages add up to 100 because of rounding.

**Figure 1:**
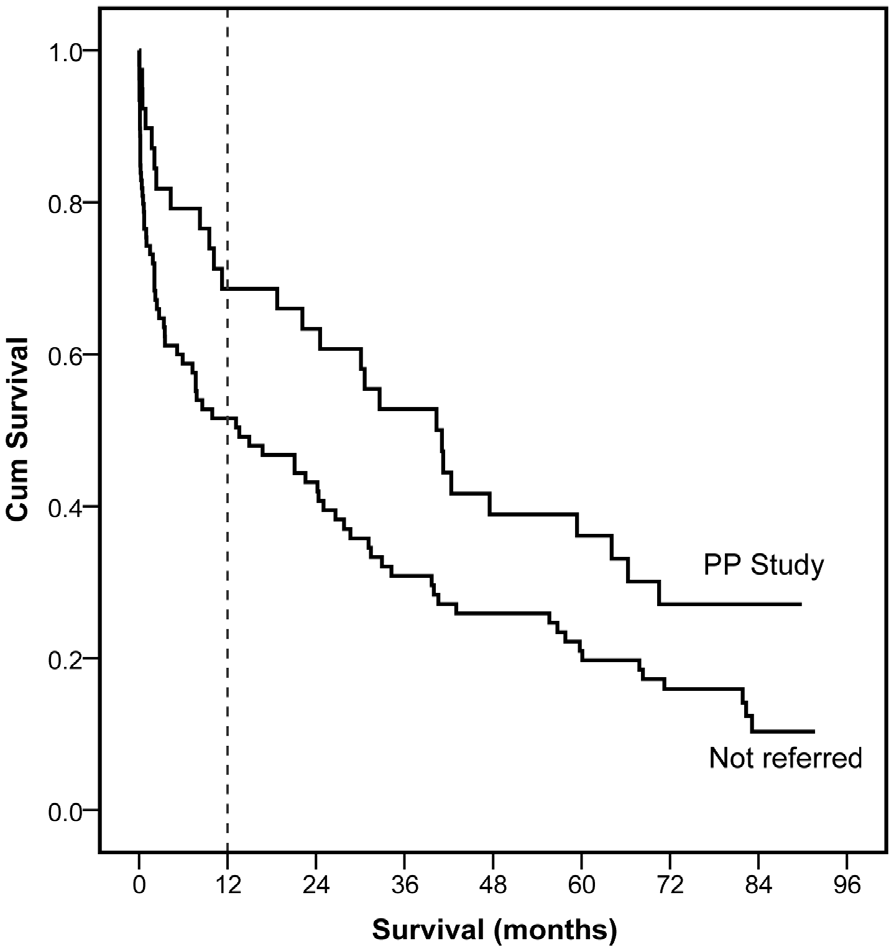
Survival of patients referred and not referred to study after lower limb amputation.

There was a significant difference in one year survival between the groups; 67% of referred patients survived compared with 40% of non-referred patients (p = 0.004). Overall survival time after amputation also differed significantly (figure 1): median (standard error) survival for referred group = 41.1 (7.9) months, non-referred = 13.6 (6.6) months, χ^2^(1df) = 5.6; p = 0.018.

To verify whether or not the differences in the groups were linked to the significantly poorer survival of the non-referred population, characteristics of patients who survived to 12 months are presented in table 2. There remained a significantly younger median (p = 0.016) and mean (p = 0.007) age difference between the referred and non-referred group. Again, the referred group were more likely to have had a knee disarticulation, less likely to have had a transtibial amputation (p = 0.041), and more frequently had amputation because of non-vascular causes (0.012). There were no other significant differences between the referred and non-referred groups in 12 month survivors. Overall survival differed by 9 months (median (standard error) survival for referred group = 64.1 (14.7) months, non-referred = 55.6 (11.5) months, χ^2^(1df) = 1.8; p = 0.177) and non-referred patients were more frequently discharged to a care center while referred patients were more often discharged to a rehabilitation center (p = 0.130).

**Table 2:** Characteristics of referred and non-referred patients who survived ≥12 months after amputation.

n (%), unless stated otherwise	Referred (PP study)		Not referred		p	Actual population	
Total included	26	(38)	43	(62)		69	
Age, median (IQR)*	65.4	(50.1; 75.3)	72.1	(65.2; 81.2)	0.016†	70.2	(61.8; 77.9)
Age, mean (sd)	62.2	(15.1)	71.3	(12.0)	0.007‡	67.9	(13.9)
Men	14	(54)	28	(65)	0.249	42	(61)
*Cause of amputation*							
Vascular	19	(73)	41	(95)	0.012	60	(87)
Other	7	(27)	2	(5)		9	(13)
*Level of amputation*							
bilateral	0	(0)	3	(7)	0.041§	3	(4)
transfemoral	8	(31)	8	(10)		18	(26)
knee disarticulation	6	(23)	2	(5)		8	(12)
transtibial	12	(46)	28	(65)		40	(58)
Peripheral vascular procedure	15	(58)	20	(47)	0.258	35	(51)
*Discharged to*							
home	8	(31)	10	(24)	0.130	18	(27)
rehabilitation centre	8	(31)	6	(14)		14	(21)
care centre	10	(39)	26	(62)		36	(53)

p is difference between referred and non-referred groups;

* Median age presented because data were not normally distributed, and mean age also presented to enable comparison to original PP Study;

† Mann Whitney U Test;

‡ Independent sample t-test; all others are Chi-square test;

§ Exact method used as cell count assumptions not met; Actual population is presented to enable comparison of characteristics, no statistical analysis was performed; not all percentages add up to 100 because of rounding.

## Discussion

A prospective study of phantom pain aimed to report all patients undergoing primary major lower limb amputation yet more than 70% of potential participants were not referred in the first year. This resulted in a sample that was younger, less likely to have had vascular related amputation and differed in both pre and post care setting than the actual population who underwent LLA [5].

The PP study described a prevalence rate for phantom pain of 32% measured 6 months after amputation [5]. Other literature measuring occurrence at 6 months has reported more than double this amount, with 65–79% of people having phantom pain [13], [14]. Occurrence rates at 6 months in trials to treat phantom pain range from 0–38% (0/10 in intervention; 5/13 in control group) [15] to 9–73% (1/11 in intervention; 8/11 in control) [16]. The PP study has a lower rate of phantom pain than expected which raises some uncertainty in generalisation and clinical application of the protective factors identified. These protective factors should be considered within the context of the biased population.

Three protective factors against the development of phantom pain were described: being male, having a lower limb amputation (versus an upper limb amputation) and time since amputation [5]. In the current study, there were no differences in sex between referred and non-referred patients (upper limb amputations were not included and only one year of the PP study was analysed). Other factors in the PP study were also investigated but not found to be significant, including level of amputation, cause of amputation and age at time of amputation. None of these factors were accurately represented by the sample referred to the PP study and it is not possible to draw a valid conclusion over their influence based only on this data.

The factors identified in the PP study were largely in disagreement to other literature. In addition to prevalence rates being much higher than the PP study, sex is reported as being unrelated to occurrence of phantom pain [13], [17] although males and females may deal with the pain differently [18]. Increasing age is shown as having a higher risk of phantom pain [19] while others have reported no relation [17] or not included age in their analysis [13]. More proximal amputation levels, and having bilateral amputation, may increase a person’s risk of phantom pain [19] although again others have found no association between the two [13], [20].

The contradictory findings surrounding phantom pain in these different populations are, at least in part, also partly attributable to differences in definitions and study design. Cut-off points for what constitutes phantom pain can include people with almost constant pain or people who experience only occasional pain [5]. Most previous studies of phantom pain are cross sectional and direct cause and effect cannot be stated. Studies include people with differing lengths of time since amputation, from a few months to many years [18], [19], [21], yet time since amputation is another factor potentially linked to phantom pain. Poor physical condition from co-morbidities and cognitive deficit leads to difficulties in patient inclusion and sample sizes are generally small. As amputation research is also characterised by a high mortality rate, follow up rates are often low. In this study almost 50% of the total population had died within 12 months of their first major amputation, including 33% of the referred group. The PP study is the largest longitudinal study of phantom pain performed (total included at first follow up was 85 from 120 included) and followed patients for up to 3.5 years [5]. Unfortunately, the substantial bias seen in the population presents a major limitation and there remains limited evidence around risk factors associated with phantom pain. Reviews looking at mechanisms and treatment of phantom limb pain reveal similar shortcomings in methodology [22]–[26].

A major difficulty with amputation outcome research is obtaining large and representative samples. The reasons for having an amputation make it difficult for many cases to be included in research as elderly people with systemic disease tend not to be considered for participation and have a higher rate of drop out or death [1]. This appeared to be a key element of (non)recruitment to the PP study, with patients who were older and with amputation due to vascular disease least likely to be referred. Data, or at least their estimates, on non-participants (including people who did not give consent, patients who are excluded, deaths and drop outs) should be communicated by authors. In the PP study, all referral sources were requested to provide this information, but unfortunately it did not occur. Minimal data presented in amputation research should include the number of participants and non-participants, age, sex, level of amputation and cause of amputation.

Our data were split to look at 12 month ‘non-survivors’ compared to ‘survivors’. With outcomes of interest for the ‘frailer’ group likely differing from the survivors, it is reasonable that they are not included in longitudinal outcome research. Unfortunately, our results showed that a substantial number of this healthier group, the 12-month survivors, also failed to be included in the PP study. In designing any study, gaining strong interest and support from relevant stakeholders and referral sources is vital. In the case of the PP study, referral sources (surgeons and staff) were informed of the aims and methodology at a regional meeting, with verbal agreements given for participation (referral of patients). The high referral rate (>85% of all within hospital, contributing >69% of all referred) from the study’s operating/base hospital, suggests either the physical presence of the investigator and/or simply being the study’s main location are the most effective strategies for recruitment. Across the entire regional network of hospitals, a physical presence was not possible. Attempts to counter this limitation through regular phone and newsletter contact were unsuccessful, with 6 hospitals not referring patients in the first year despite their agreement. Improving recruitment via clinicians is a difficult task; even large randomised controlled trials have great difficulty identifying successful strategies [27]. Adding to this are strict timeframes of the inclusion criteria of the PP study with referral set for within 5 days. This meant that surgeons (and their staff) were primarily responsible for identifying cases, at a time when other factors, such as pre-operative assessment, can naturally be of a greater priority. It is not routine practice for the rehabilitation physician to be involved pre-surgically so this additional referral source was not utilised. Other alternatives were not considered as clinically relevant options, such as extending the inclusion period to >5 days, as this would have introduced problems with recall.

### Limitations

The population study used data from a retrospective review of medical files and as such, information was limited to what is included in these. The data were collected for a concurrent study on incidence and as such, they are considered to be complete. However, we acknowledge that cases may have been missed. If anything, the sample is an underestimation, although we do not expect that this would have any large affect on our main findings. Another limitation from the study design (review of medical records) is not having access to information on disease severity or duration of disease. Further, unless it is of a very severe nature, cognitive status is infrequently noted in the medical files. However, this is likely to be a major source of selection bias in LLA research given that vascular disease affects the body systemically. Finally, there was no information on survival status available for 27 patients and our results are likely to be an underestimate of survival time.

In the current study, all patients referred to the PP study were considered as one group. However, 16 (41%) of these patients were not part of the analyses as they did not meet the criteria for inclusion. These excluded patients were older and more likely to have had amputation because of vascular disease than included patients [5]. The findings of this current study should therefore be considered as a conservative estimate of the impact of selection bias as these excluded older patients with vascular disease remained within the ‘referred’ group.

### Conclusion

Selection bias is common, and perhaps inherent, in amputation research. Over 70% of patients were missed in a study of phantom pain, resulting in a younger population who were less likely to have had vascular related amputation and differed in respect to their pre- and post-care setting. As a result, phantom pain was possibly underestimated and the resultant protective factors identified should be considered only within the context of the biased population. Two important elements for improving research into amputation outcomes were identified: (a) failure to refer relevant cases (recruitment bias); and (b) failure to communicate reasonable non-inclusions. Potential bias should be more clearly presented by authors and subsequent conclusions and clinical decisions made with greater caution. In addition, maximum efforts should be directed to research methodology which minimises the influence of bias.
